# Simulation and Experimental Study of the Single-Pulse Femtosecond Laser Ablation Morphology of GaN Films

**DOI:** 10.3390/mi16010085

**Published:** 2025-01-13

**Authors:** Mingyuan Wang, Tong Zhang, Yanping Yuan, Zhiyong Wang, Yanlei Liu, Lin Chen

**Affiliations:** 1 School of Physics and Optoelectronic Engineering, Beijing University of Technology, Beijing 100124, China; 2202283133@stu.htu.edu.cn (M.W.); ypyuan@bjut.edu.cn (Y.Y.); zywang@bjut.edu.cn (Z.W.); lnlychen@emails.bjut.edu.cn (L.C.); 2 Henan Key Laboratory of Infrared Spectrum Measures, Applications College of Physics, Henan Normal University, Xinxiang 453007, China; 2018150@htu.edu.cn

**Keywords:** femtosecond laser, GaN film, multiphysics model, ablation morphology

## Abstract

Gallium nitride (GaN) exhibits distinctive physical and chemical properties that render it indispensable in a multitude of electronic and optoelectronic devices. Given that GaN is a typical hard and brittle material that is difficult to machine, femtosecond laser technology provides an effective and convenient tool for processing such materials. However, GaN undergoes complex physical and chemical changes during high-power ablation, which poses a challenge to high-precision processing with controllable geometry. In this study, the quantitative relationship between the parameters of a single-pulse femtosecond laser and GaN ablation morphology was investigated using isotherm distribution. A multiphysics model using COMSOL Multiphysics^®^ was developed to generate the isothermal distributions. Experiments were conducted on the femtosecond laser ablation of GaN at various single-pulse energies, and the resulting ablation morphologies were compared with the predictions from the multiphysics model. The comparison demonstrated that the calculated isotherm distribution accurately predicted not only the ablation diameter and depth but also the crater shape across a broad range of laser fluences. The predicted errors of the ablation diameters and depths were within 4.71% and 10.9%, respectively. The root mean square error (RMSE) and coefficient of determination (R^2^) were employed to evaluate the prediction errors associated with the crater shapes, which fell within the range of 0.018–0.032 μm and 0.77–0.91, respectively. This study can provide an important reference for utilizing femtosecond lasers in the precise ablation of GaN to achieve desired geometries.

## 1. Introduction

GaN is characterized by a wide bandgap and superior material properties, as evidenced by a high breakdown electric field, high operating frequency, high charge saturation speed, and high chemical stability [1]. In recent years, GaN has been utilized in widespread applications, including light-emitting diodes [2,3], microelectromechanical devices [4], vertical-cavity surface-emitting lasers (VCSELs) [5] and solar cells [6]. In the fabrication of GaN devices, effective methods for processing GaN at the sub-micron/micron scale are required [7,8,9]. However, GaN is a typical hard and brittle material that is difficult to machine. Femtosecond lasers are characterized by their exceptionally short pulse durations and high peak powers, which provide a powerful and advantageous tool for processing such materials. Currently, femtosecond lasers have been employed for the processing of GaN materials [10,11,12,13]. Nevertheless, materials and femtosecond lasers interact in a complicated, nonlinear, non-equilibrium process that involves various physical processes, including energy absorption, energy transfer between electrons and lattices [14], plasma generation [15], and material phase change and removal [16]. Additionally, GaN is subject to thermal decomposition and oxidation when the temperature is above 1223 K [17,18], further complicating the laser–material interaction mechanism. In this regard, it could be a challenging task to achieve the high-precision controllable processing of GaN. The determination of the optimal processing parameters frequently requires a large number of experiments, which can consume a significant amount of time and resources.

Modelling is an efficient and convenient approach for revealing the quantitative correspondence between laser parameters and ablation morphology features. Several studies have been carried out on modelling the interaction between femtosecond lasers and semiconductors and dielectric materials that have few free electrons. In addition, relevant experiments have been conducted that have validated the predicted ablation morphology. For the prediction of ablation morphology, various methods have been developed. Jiang et al. [19,20] employed critical electron density as the ablation condition to simulate the depth and morphology of ablation in barium aluminium borosilicate. The calculated ablation depths were found to be consistent with previously published experimental data. Subsequently, the ablation depth and ablation morphology of fused silica were calculated according to the distribution of free electron density and lattice temperature, respectively. The ablation morphology calculated by the two methods was found to be consistent, and the calculated ablation depths were consistent with the previously published experimental data. Guizard et al. [21] assumed that the pertinent threshold parameter is the quantity of energy absorbed at the conclusion of the action of a femtosecond laser pulse. They conducted simulations of the ablation crater depth of α-Al_2_O_3_ and made comparisons with experimental data. They found that accounting for the accumulation of laser energy at the surface and its propagation through the sample resulted in a superior data fit at higher laser energy densities. Chen et al. [22] and Yang et al. [23] conducted simulations to predict the ablation depth and diameter of monocrystalline silicon and SiC with the isotherm determined as the ablation condition, respectively. It was found that the simulation with the experimental results revealed greater consistency in the middle-fluence regime.

However, the best method for predicting the femtosecond laser ablation morphology of GaN is still uncertain. There are not many existing theoretical studies on the interactions between femtosecond lasers and GaN. Wang et al. [24] calculated the change in surface reflectivity and electron–lattice temperature after laser irradiation, but did not offer any predictions about the ablation morphology. Cai et al. [25] further computed the progression of free electron density at a single power density. The resulting ablation morphology was then predicted at this power density based on the critical electron density but without experimental validation. It would appear that there is still a dearth of accurate prediction methods and experimental validation for the geometric and morphological characteristics of GaN subjected to femtosecond laser ablation. Moreover, the established modelling of the femtosecond laser–material interaction usually focuses on the prediction of ablation diameter and depth and less on the geometry profile. Although the ablation diameter and depth from experiments and simulations are in agreement, the ablation profiles may not be identical.

In this study, the quantitative relationship between the parameters of a single-pulse femtosecond laser and GaN ablation morphology was investigated using isotherm distribution. A multiphysics model was established using COMSOL Multiphysics^®^ 4.3 to generate the isothermal distributions. The simulation took into account the variation in temperature field and carrier density, as well as the dynamic optical properties of the material. The single-pulse femtosecond laser ablation morphology was predicted by isothermal distributions. To validate the model, a series of femtosecond laser ablation experiments of GaN at different energies were carried out. It is noteworthy that this study not only compares the measured and simulated ablation diameter and depth but also performs a quantitative evaluation of the predicted crater shapes. Moreover, as GaN devices become smaller and more integrated, there is a need for greater processing accuracy and resolution, often requiring a reduction in spot size. Therefore, the spot radius used in this study falls within the micrometre scale, which is smaller than previous modelling studies. The threshold for a single-pulse femtosecond laser with a micrometre spot radius ablating GaN was simulated for different pulse widths.

## 2. Modelling and Simulation

### 2.1. Principle Description

When a femtosecond laser interacts with GaN, the transient density of free electrons grows considerably, which in turn influences the subsequent process. It is therefore necessary to establish the free electron density. The Fokker–Planck equation is being used for the purpose of simulating the free electron density *n_e_* with the consideration of multi-photon absorption, carrier diffusion, and Auger recombination [20,26].

(1) $$\frac{\partial n_{e}}{\partial t}=\nabla (D\nabla n_{e})+\alpha_{i}In_{e}+\frac{\delta_{N}}{N\hbar \omega }I^{N}-\frac{n_{e}}{\tau }$$

where *I* is the laser intensity, *τ* is the free electron decay constant, *α_i_* stands for the impact ionisation coefficient, *δ_N_* is the N-photon cross-section coefficient, and *ħω* is the photon energy. The diffusion coefficient *D* is defined as *D = k_B_T_e_u_e_*/*e*, where *k_B_* and *T_e_* stand for the Boltzmann constant and the electron temperature, and *u_e_* and *e* represent the electron charge and electron mobility. The number of photons required to free a bound electron is determined by the ratio of the material bandgap to the photon energy [27,28]. In this study, the three-photon absorption of GaN was considered, given that the laser wavelength used was 800 nm (1.55 eV) [29].

After exposure to a femtosecond laser pulse, the bound electrons within the GaN material experience excitation in large quantities, resulting in the transient density of free electrons increasing considerably. The optical properties of the material undergo a transformation as a consequence. In accordance with the Drude model, the dielectric function is defined by the following expression [30]:

(2) $$\varepsilon =\varepsilon_{1}+i\varepsilon_{2}=1+(\frac{n_{e}e^{2}}{m_{e}\varepsilon_{0}})(\frac{-{\tau_{e}}^{2}+i\tau_{e}/\omega }{1+\omega^{2}{\tau_{e}}^{2}})$$

where *ε* represents the complex dielectric function, while *ε*_1_ and *ε*_2_ represent its real and imaginary components, respectively; *m_e_* represents the mass of an electron; *ω* stands for the laser frequency; and *τ_e_* denotes the free electron relaxation time.

The complex refractive index *f* can be expressed as

(3) $$f=n+ik=\sqrt{\varepsilon }$$

in which *n* and *k* represent the refractivity and the extinction coefficient. Therefore, the following functions can be obtained:

(4) $$n=\sqrt{\frac{\varepsilon_{1}+\sqrt{{\varepsilon_{1}}^{2}+i{\varepsilon_{2}}^{2}}}{2}}$$

(5) $$k=\sqrt{\frac{-\varepsilon_{1}+\sqrt{{\varepsilon_{1}}^{2}+i{\varepsilon_{2}}^{2}}}{2}}$$

The Fresnel expression provides a means of calculating the GaN surface reflectance

(6) $$R=\frac{{\left(n-1\right)}^{2}+k^{2}}{{\left(n+1\right)}^{2}+k^{2}}$$

The total absorption coefficient of laser energy is derived as

(7) $$\alpha =\frac{2\omega k}{c}+\alpha_{i}n_{e}E_{g}$$

in which *E_g_* is the bandgap of GaN.

The two-temperature model (TTM) is employed for the purpose of describing the process of electron–lattice interactions [20].

(8) $$C_{e}(T_{e})\frac{\partial T_{e}}{\partial t}=\nabla (k_{e}(T_{e})\nabla T_{e})-G(T_{e}-T_{l})+S$$

(9) $$C_{l}(T_{l})\frac{\partial T_{l}}{\partial t}=G(T_{e}-T_{l})$$

where *G* represents the electron–lattice coupling factor, *S* denotes the laser source term, *C_e_* and *C_l_* stand for the specific heat of the electron and lattice, respectively, *T_l_* represents the lattice temperature, and *k_e_* represents electron conductivity. Equation (1) is the free electron density equation which describes the temporal and spatial variations in free electron density. These variations influence the material’s optical properties, such as reflectivity and the absorption coefficient. These changes are captured through Equations (2)–(6) that link free electron density to the dielectric constant and reflectivity, as well as the absorption coefficient as shown in Equation (7). Consequently, the altered reflectivity and absorption coefficient modify the laser intensity distribution within the material, as depicted in Equation (11). The distribution is then coupled to the TTM via the laser source term *S*, which represents the energy input from the laser.

It is assumed that the original laser intensity, both in terms of time and space, follows a Gaussian distribution prior to interaction with the material [22,31]

(10) $$I_{0}=\sqrt{\frac{4\ln2}{\pi }}\frac{F_{0}}{t_{p}}\exp(-\frac{2r^{2}}{{r_{0}}^{2}}-\frac{4\ln2{\left(t-2t_{p}\right)}^{2}}{t_{p}^{2}})$$

where *F*_0_ is the peak laser energy density, *t_p_* represents the laser pulse width, and *r*_0_ is the laser spot radius. The distribution of laser intensity within GaN can be defined as

(11) $$I=I_{0}(1-R)\exp(-\int_{0}^{z}\alpha dz)$$

The laser source term can be illustrated as follows:

(12) $$S=\frac{\partial I}{\partial z}=-\alpha I=-\frac{2\omega k}{c}I-\alpha_{i}In_{e}E_{g}$$

The TTM subsequently describes the energy exchange between the electrons and the lattice, providing a comprehensive understanding of the thermal and optical dynamics under femtosecond laser irradiation. The integration of free electron density into the TTM is essential for accurately modelling the interaction of femtosecond lasers with materials.

### 2.2. Simulation Details

On the basis of the above theory, COMSOL Multiphysics^®^ was used to establish the multiphysics model. As the femtosecond laser is an axisymmetric Gaussian beam, it is possible to reduce the model to a two-dimensional rotational model without affecting the precision of the calculations, thereby reducing the computational burden and conserving computational resources. Generally, the grown GaN film has a thickness of nanometres to micrometres. Accordingly, the dimensions of the established model are defined as a radius of 4 μm and a thickness of 2 μm. The COMSOL Multiphysics^®^ heat transfer module is employed for the resolution of two-temperature equations, while the partial differential equations module is employed for the solution of the free electron density equation and the heat source equation. Since the ablation process of GaN occurs on a femtosecond timescale, it can be reasonably assumed that the heat exchange process with the external environment can be disregarded. Therefore, the unmachined surfaces are set as adiabatic boundary conditions.

**Figure 1:**
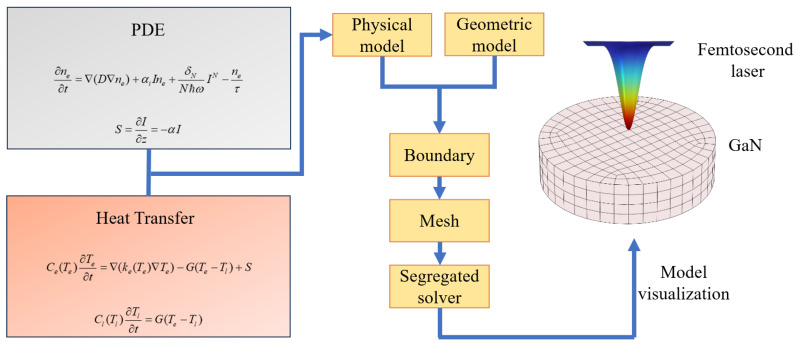
Flowchart of the modelling process.

**Figure 2:**
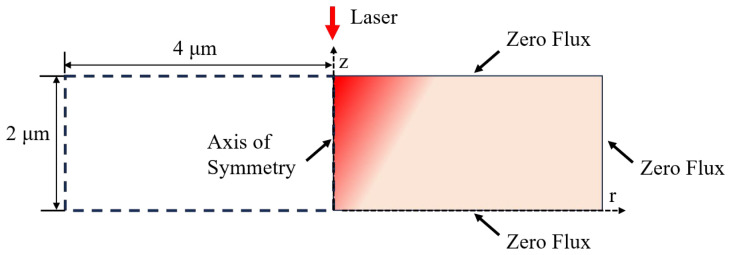
Applied boundary conditions for modelling.

The overall modelling process is illustrated in Figure 1, and the boundary conditions are defined as shown in Figure 2. The mesh is configured as a free triangular mesh, with the overall maximum cell size set to 0.022 μm and the minimum cell size set to 0.1 nm. Furthermore, the mesh at the surface of the material is refined to a maximum cell size of 1.8 nm, with the objective of enhancing the calculation’s precision.

### 2.3. Model by COMSOL Multiphysics^®^

**Figure 3:**
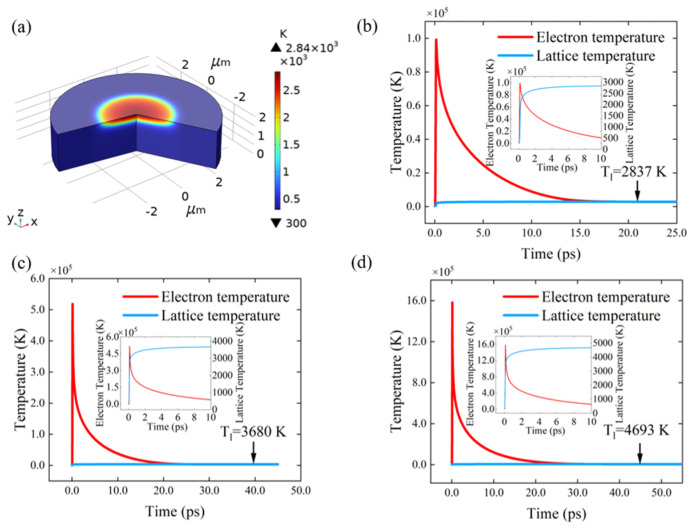
Evolution of the temperature at the surface of GaN. (**a**) Lattice temperature distribution of a geometric model with an 8 μm diameter and 2 μm thickness cylinder at a single-pulse energy of 0.08 μJ. The electron and lattice temperature variation, with an enlarged view within 10 ps (inset), at single-pulse energies of (**b**) 0.08 μJ (**c**), 0.6 μJ and (**d**) 1.8 μJ.

Table 1 enumerates the GaN parameters utilized in the multiphysics model. The laser parameters were set to a pulse duration of 50 fs, a beam size of 1.62 μm, and a wavelength of 800 nm. The lattice temperature distribution of GaN subjected to ablation using a single-pulse energy of 0.08 μJ is illustrated in Figure 3a. As illustrated in Figure 3b, the electron and lattice temperatures in the centre of the laser irradiation zone of the developed model were selected for investigation with a view to determining how they change during femtosecond laser irradiation. In the initial phase of laser irradiation, the electron temperature exhibited a rapid increase, which was followed by the transfer of energy to the lattice through electron–phonon scattering. This resulted in a gradual increase in the lattice temperature, which eventually reached an equilibrium state at 2837 K. At this time, the lattice temperature already exceeded the decomposition temperature of GaN (1223 K) and the melting temperature (1973 K) [32]. GaN will decompose as follows:

2GaN(s) → 2Ga(l) + N_2_(g)

Although the single-pulse energy is quite low, the energy density is relatively high due to the spot radius used in this study being on a micrometre scale. Therefore, the lattice temperature has already exceeded the melting temperature. An increase in the size of the light spot allows for the attainment of a lattice temperature that is below the melting point [24]. To gain further insight into the impact of laser fluence, Figure 3c,d illustrate the variations in electron and lattice temperatures at single-pulse laser energies of 0.6 and 1.8 μJ, respectively. The lattice temperatures reach 3680 and 4693 K under thermal equilibrium, respectively. It is evident that the surface temperature of GaN increased in conjunction with the laser fluence.

**Table 1:** Parameters of GaN utilized in the multiphysics model [24,29,30,33,34].

Parameter	Symbol	Value
Boltzmann constant	*k_B_*	1.380649 × 10^−23^ (J·K^−1^)
Electron mobility	*u_e_*	800(T_l_/300)^−1.5^ (cm^2^·V^−1^·s^−1^)
Bandgap energy	*E_g_*	3.39 (eV)
Multiphoton absorption coefficient	*δ*_3_	0.011 (cm^3^·GW^−2^)
Electronic constant volume heat capacity	*C_e_*	70T_e_ (J·m^−3^·K^−1^)
Electronic thermal conductivity	*k_e_*	220T_e_/T_l_ (W·m^−1^·K^−1^)
Original valence electron density	*n*_0_	2.8 × 10^23^ (m^−3^)

## 3. Experimental

**Figure 4:**
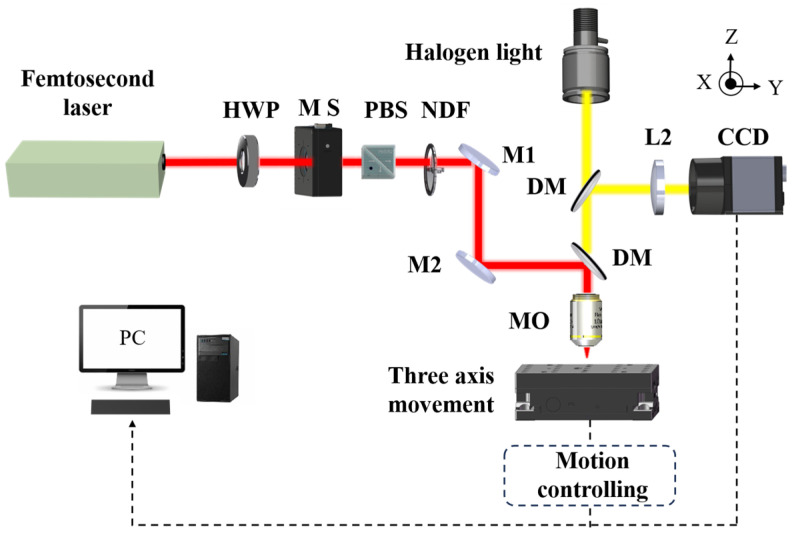
Schematic of the femtosecond laser processing experimental setup.

The materials used in this experiment comprised 2-in C-face (0001) undoped GaN grown on a C-face sapphire (0001) substrate. The substrate had a thickness of 430 μm. The GaN layer had a thickness of 4.5 μm and mobility of higher than 300 cm^2^/Vs. The schematic representation of the experimental setup for femtosecond laser processing is presented in Figure 4. A femtosecond laser (Spitfire ACE PA401K, Newport Spectra-Physics, MKS Instruments Inc., Andover, MA, USA) with a wavelength of 800 nm, a pulse width of 50 fs (FWHM), an adjustable repetition rate from 1 to 1000 Hz, and a maximum single-pulse energy of 12 mJ was employed in this study. To ensure the continuous adjustment of the laser pulse energy, a half-wave plate (HWP), a polarising beam splitter (PBS), and a neutral density filter (NDF) were employed. The exposure time could be adjusted by a mechanical shutter (MS). Two mirrors (M1 and M2) and a dichroic mirror (DM) were employed to direct the laser into a microscope objective (MO), which served to focus the beam onto the material. The material was placed on a high-precision three-axis movement stage (Newport, MKS Instruments Inc., Andover, MA, USA). The femtosecond laser ablation process was monitored and documented using a CCD camera.

Specific single-pulse energies were chosen for different fluence regimes: low fluence (0.08–0.4 μJ), middle fluence (0.6–1.59 μJ), and high fluence (1.8–4.04 μJ). The correspondence between single-pulse energies and laser fluences for these regimes was established as follows: low fluence (1.94–9.74 J/cm^2^), middle fluence (14.52–38.54 J/cm^2^), and high fluence (43.57–97.79 J/cm^2^).

## 4. Results and Discussion

### 4.1. Experimental Ablation Threshold and Morphology

Femtosecond laser ablation experiments were conducted on GaN at varying single-pulse energies. By regulating the scanning velocity of the movement stage, a row of ablation craters was obtained for each single-pulse energy. Each crater was separated by approximately 40 μm to prevent interference between ablations. The ablation craters were observed and measured using scanning electron microscopy (SEM, FE-STEM SU9000, Hitachi, Ltd., Tokyo, Japan) and confocal laser scanning microscopy (CLSM, OLYMPUS-LEXT-OLS 4000, OLYMPUS, Tokyo, Japan).

The results of the ablation process for each parameter of the femtosecond laser were obtained by averaging the diameter and depth of multiple ablation craters. The D^2^ method, as proposed by Liu [35], was employed to investigate the relationship between the ablation crater diameter squared and the single-pulse energy density. In this experiment, the energy of the pulsed laser followed a Gaussian distribution and its energy distribution satisfied the following relationship:

(13) $$\varphi_{h}=\varphi_{0}\exp(\frac{-D^{2}}{2\omega_{0}^{2}})$$

where *φ_h_* represents the ablation threshold energy density, *D* stands for the material removal diameter, and *ω*_0_ represents the beam radius. Hence, the following equation can be obtained:

(14) $$D^{2}=2\omega_{0}^{2}\ln(\frac{\varphi_{0}}{\varphi_{h}})$$

**Figure 5:**
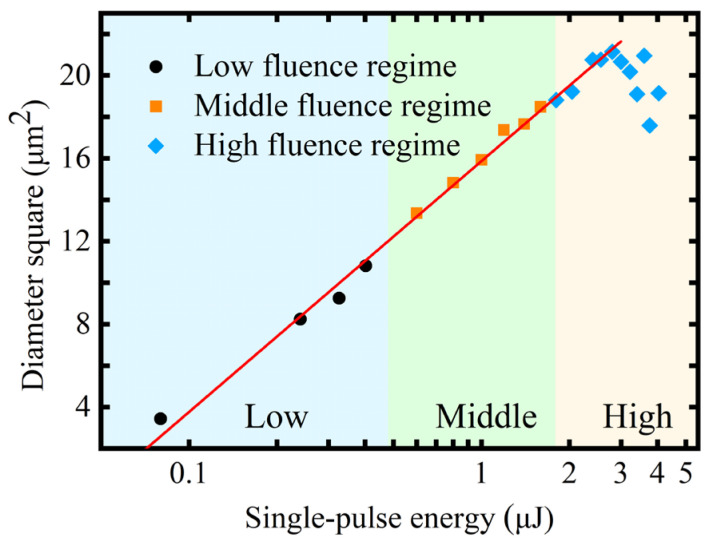
Relationship between the ablation crater diameter squared and the single-pulse energy.

**Figure 6:**
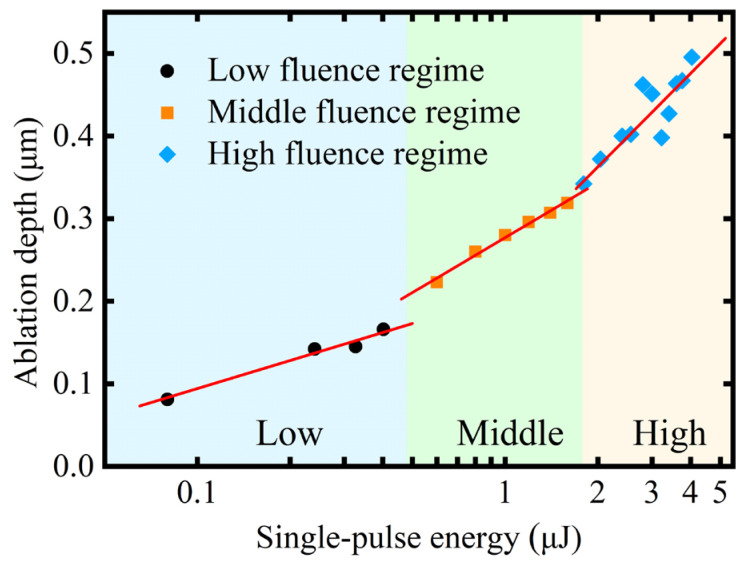
Relationship between the ablation crater depth and the single-pulse energy.

Figure 5 demonstrates the dependence of the ablation diameter squared on the logarithm of laser energy density. At a single-pulse energy below 3 μJ, the ablation diameter exhibited a linear increase as the laser energy increased. At higher laser energies, the observed ablation diameter was affected by the flow of molten materials and did not exhibit linear growth. Thus, only the data before 3 μJ were used in the fitting of Equation (14). The fitted line indicates that the actual beam radius was 1.62 μm, with a theoretical ablation threshold of 1.19 J/cm^2^. The threshold fluence calculated in this study was higher than the previous studies. This can be attributed to the smaller beam radius after focusing via the microscope objective in this experiment. The ablation threshold was influenced by the beam radius, which inversely correlated with the decrease in beam radius [36,37]. The dependence of the ablation crater depth on single-pulse energy is shown in Figure 6. With 0.4 and 1.59 μJ as the energy boundaries, differences in growth slopes were observed, which can be divided accordingly into three regimes, namely, low-fluence, middle-fluence, and high-fluence regimes.

**Figure 7:**
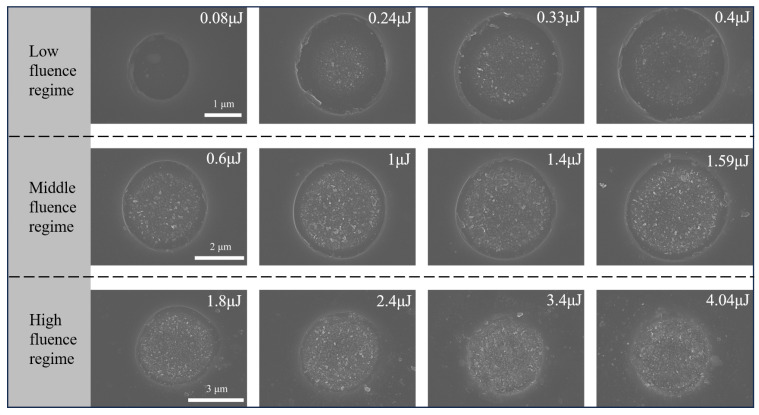
Ablation morphology of GaN ablated by single-pulse laser energy (from 0.08 to 4.04 μJ) observed by SEM and subdivided into low-fluence, middle-fluence, and high-fluence regimes.

The removal mechanism of materials in femtosecond laser ablation is related to laser parameters and material properties, including Coulomb explosion, photomechanical fragmentation, melting and phase explosion. In addition, the removal of GaN materials is influenced by thermal decomposition and bubble nucleation. Figure 7 illustrates the morphology of the ablation crater within a wide range of laser fluences from 0.08 to 4.04 μJ. According to the different morphologies, it can be divided into three fluence regimes, corresponding to the regimes given in Figure 6. One or more of these mechanisms were involved in these regimes.

In the low-fluence regime, the ablation craters exhibited a regular shape, presenting a round and shallow configuration. The diameter and depth of these craters increased linearly with the logarithm of the single-pulse energy. When an ultrafast pulsed laser irradiates on the surface of GaN, the free electrons are rapidly excited [20]. Upon reaching the critical free electron density, a Coulomb explosion ensues [38]. A clear boundary was evident at the periphery of the crater, resulting from photomechanical fragmentation [39]. According to the simulation results, the lattice temperature of GaN at this fluence regime has already exceeded its decomposition and melting temperatures. As a result of the N_2_ produced by GaN decomposition, along with the mechanical stress exerted on the surface by rapidly expanding gas, a layer of fragments detached. In addition, N_2_ could explain the slight protrusion observed in the centre of the crater. The N_2_ bubbles beneath the surface caused surface deformation, which gave rise to a slight bulge [24]. A comparable phenomenon was observed in CaF_2_ [40,41].

In the middle-fluence regime, more laser energy was absorbed, resulting in a gradual enhancement of the decomposition of GaN. The ablation depth increased at a faster rate. The simulation results indicated a rise in the lattice temperature to 3680 K, which caused bubbles to nucleate inhomogeneously in the melting layer. Meanwhile, the breakdown of the Ga–N bond promoted the formation of Ga, which melted at a high temperature and eventually solidified in the crater to form sub-micron particles [24,42]. As the laser fluence increased, the number of nanoparticles in the crater resulting from the Coulomb explosion and the material fragments generated by photomechanical fragmentation exhibited a corresponding increase. Some of these nanoparticles were ejected from the crater and sputtered to the outer edge.

In the high-fluence regime, a large amount of plasma and material fragments were splattered outside the crater. The primary phenomenon was thermal ablation, resulting in phase explosions when the bubble radius reached its critical size [24]. Melting droplets and other substances within the crater were splattered out by the recoil force generated by the phase explosion. It is evident from Figure 7 that a large quantity of splattering traces and material fragments are present in the vicinity of the crater. As molten materials flowed towards the edges, solidification followed, resulting in a crater that has almost no clear edges. The diameter of the ablation crater was found to decrease as a result of the molten materials solidifying inside the crater, which also affected the measurement of the ablation depth. Furthermore, the morphology of the ablated GaN was influenced by the combined effects of the Coulomb explosion, photomechanical fragmentation, phase explosion, material melting and vaporisation [39]. In this fluence regime, the quality of the machining was not ideal. If the ablation morphology is to be regular, either high-power fluence should be avoided or molten material should be cleaned and removed by chemical etching or other means after ablation.

### 4.2. Ablation Morphology Prediction and Verification

**Figure 8:**
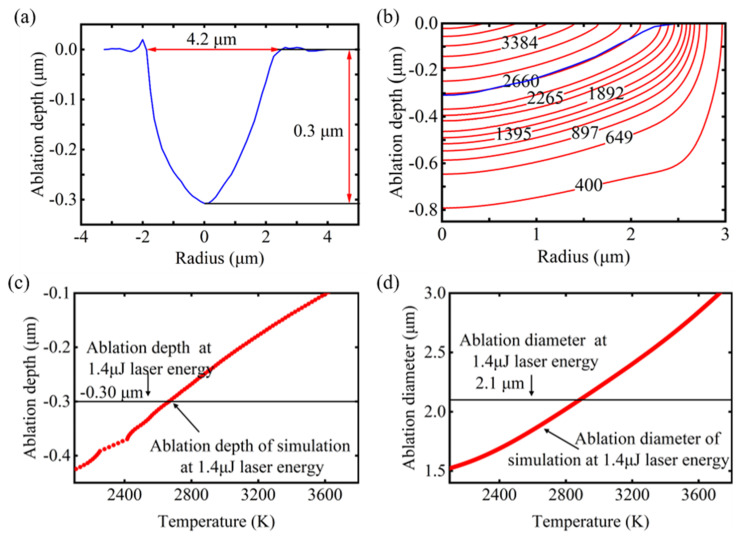
Experimental and simulation results of 1.4 μJ single-pulse laser ablation of GaN: (**a**) GaN ablation crater shape measured by CLSM. (**b**) Simulated isotherm distribution of different lattice temperatures (red line) and GaN ablation crater shape measured by CLSM (blue line). (**c**) Comparison of simulation ablation depth at varying temperatures with experimental results. (**d**) Comparison of simulation ablation diameter at varying temperatures with experimental results.

In the field of thermodynamics, the removal of GaN can typically be assumed based on lattice temperature: the phase change and thermal decomposition occur if the lattice temperature exceeds a specific threshold. When it comes to the femtosecond laser ablation of GaN, according to the phase-explosive mechanism and the Coulomb explosion mechanism, the lattice temperature can still be employed to determine the removal of material [20,43]. In the verification experiment pertaining to the ablation condition, the single-pulse energy of 1.4 μJ was chosen for investigation within the middle-fluence regime. The ablated morphology was observed using SEM and CLSM. As shown in Figure 8a, the measured ablation depth was 0.3 μm and the ablation diameter was 4.2 μm. Figure 8b illustrates the simulated isotherm distribution of various lattice temperatures of GaN following single-pulse laser ablation with 1.4 μJ, in comparison to the observed ablation morphology in the experiment. The contour of the isotherm at 2660 K exhibited the greatest similarity to the ablation morphology. The slight deviation that appeared at the edge of the ablated crater can be attributed to the fragmentation of the material. In addition, as illustrated in Figure 8c,d, the simulated ablation depth and diameter at 2660 K both exhibited a strong agreement with the experimental results. Therefore, the isotherm at 2660 K was chosen as the boundary for ablation, and any parts exceeding this temperature were considered to have been removed through ablation.

**Figure 9:**
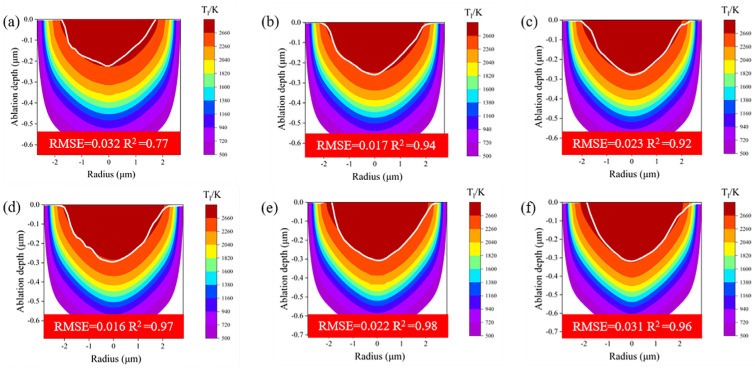
Comparison of the experimental and simulated ablation crater shapes in the middle-fluence regime. The deep-red region corresponds to the area where the lattice temperature exceeds 2660 K, while the white line represents the ablation profile measured by CLSM. (**a**) 0.6, (**b**) 0.8, (**c**) 1, (**d**) 1.19, (**e**) 1.4, and (**f**) 1.59 μJ.

Since a single-pulse energy of 1.4 μJ in the middle-fluence regime was chosen in the ablation condition verification experiment, a series of simulations were first conducted in this fluence regime with the isotherm of 2660 K as the ablation condition and compared with crater shapes measured by CLSM. In this study, the accuracy of the prediction was assessed using the commonly used model evaluation metrics of root mean square error (RMSE) and coefficient of determination (R^2^). As illustrated in Figure 9, the simulated ablation morphology in the middle-fluence regime was found to be in close agreement with the experimental crater shape (white line). The RMSE range was 0.016–0.032 μm, and the R^2^ range was 0.77–0.98.

**Figure 10:**
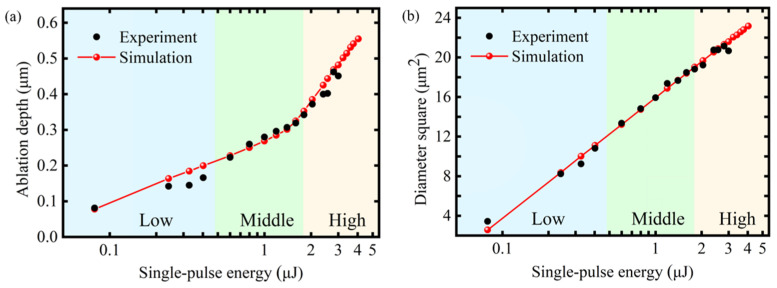
Experimental and simulated results of ablation crater size under 2660 K isothermal ablation conditions. (**a**) Comparison of the ablation crater diameter squared. (**b**) Comparison of the ablation crater depth.

On the basis of this model and the ablation conditions of the 2660 K isotherm, the ablation results across a wide laser fluence range used in the experiment, which ranged from 0.08 to 4.04 μJ, were simulated. A comparison of the modelling and experimental data for the ablation crater depth and diameter squared is presented in Figure 10a,b. In the middle-fluence regime, the simulation results closely matched the experimental results, and the simulation error for the ablation crater diameter was controlled within the range of 0–1.43%, while the error for the depth of the ablation crater was controlled within the range of 1.85–4.06%. In the low- and high-fluence regimes, the simulated results of the ablation diameter aligned with the experiment results. Nevertheless, there was a discrepancy observed between the simulated and experimental ablation depth. The simulation error for the ablation crater diameter was within the range of 0.11–13.4%, indicating that the simulated diameter was consistent with the experimental results over a wide fluence range. The simulation error for the depth of the ablation crater ranged from 1.46% to 27.26%. As depicted in Figure 10a, in the low- and high-fluence regimes, the simulation ablation depth was higher than the experimental results. One potential explanation for the discrepancy in the simulated and actual ablation depths is the absence of hydrodynamics in the model. This includes the omission of the diffusion of GaN decomposition gas and the diffusion of molten materials. In the low-fluence regime, the decomposition of GaN during the laser ablation process was relatively weak, resulting in the generation of a small quantity of N_2_. This was not conducive to the removal of molten materials, which ultimately led to a smaller actual crater depth. In addition, the simulation model did not consider fluctuations in laser output power under the low-fluence regime, which could potentially impact the ablation depth. The focusing conditions of the femtosecond laser beam can also be affected by the optical elements employed. In the high-fluence regime, the solidification of molten materials at the bottom of the crater would also impact depth measurement, resulting in a measured depth that was shallower than the actual depth. Consequently, the simulation depth was slightly larger than the experimental results in these two regimes. Moreover, once laser ablation reaches a certain depth, the energy cannot be transmitted vertically without diffusing into the adjacent areas. The saturation depth of single-pulse femtosecond laser ablation was not taken into account in the simulation. Thus, the prediction of ablation depth was more challenging than that of diameter.

**Figure 11:**
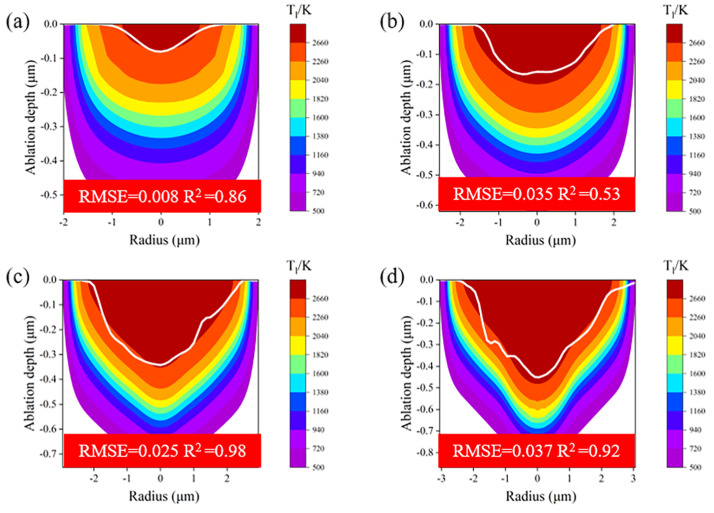
Comparison of experimental ablation morphology and simulation ablation morphology in the low- and high-fluence regimes. The region of deep red represents the area in which the lattice temperature exceeds 2660 K, and the white line is the ablation contour measured by CLSM. (**a**) 0.08, (**b**) 0.4, (**c**) 1.8, and (**d**) 3 μJ.

Further comparisons between the simulated and experimental crater shapes in both low- and high-fluence regimes were carried out. As illustrated in Figure 11, although the prediction error in ablation diameter and depth increased compared with the middle-fluence regime, the deviation of the predicted crater shape was not significant, with an RMSE within the range of 0.008–0.037 μm and R^2^ within the range of 0.53–0.98. Consequently, despite fluid dynamics not being considered in this model, the simulation results remained accurate for a broad fluence range.

**Figure 12:**
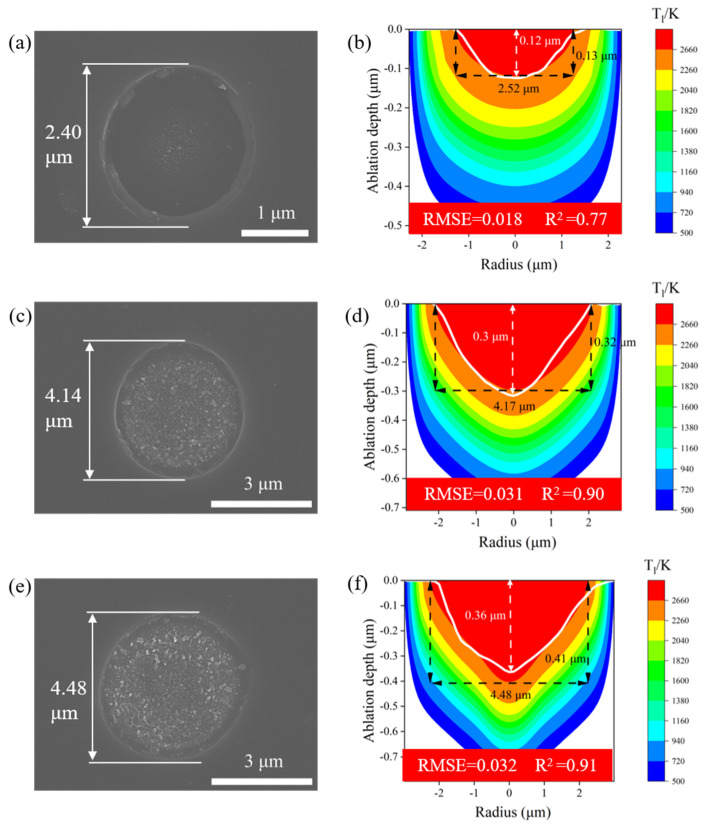
Experimental verification results and predictive results of the multiphysics model for the single-pulse laser ablation of GaN under three fluence regimes. (**a**) SEM image of the low-fluence regime (0.16 μJ). (**b**) Low-fluence regime experimental ablated crater shape and simulated results. (**c**) SEM image of the middle-fluence regime (1.32 μJ). (**d**) Middle-fluence regime experimental ablated crater shape and simulation results. (**e**) SEM image of the high-fluence regime (2.2 μJ). (**f**) High-fluence regime experimental ablated crater shape and simulation results.

To confirm the contribution of the model to experiments, femtosecond laser ablation experiments were conducted with single-pulse laser energies of 0.16, 1.32, and 2.2 μJ, corresponding to low-, middle-, and high-fluence regimes, respectively. And for all three fluence regimes, the simulated temperature distribution field at 50 ps was selected for comparison with the experimental results. This time point was chosen because it is the point at which the electron lattice temperature is in equilibrium due to the effect of different laser fluences, allowing for a more accurate comparison with the experimental data. As illustrated in Figure 12a,b, the experimental and predicted results for the diameter and depth of the ablation crater were 2.40 μm, 2.52 μm, and 0.12 μm, 0.13 μm, respectively. The results demonstrated that the prediction errors of the ablation crater diameter and depth with the single-pulse energy of 0.16 μJ were 4.71% and 7.7%, respectively. Additionally, the RMSE and R^2^ for the predicted crater shape were 0.018 μm and 0.77, respectively. According to Figure 12c,d, the experimental and predicted results for the diameter and depth of the ablation crater were 4.14 μm, 4.17 μm, and 0.32 μm, 0.30 μm, respectively. The results implied that the prediction errors of the ablation crater diameter and depth with the single-pulse energy of 1.32 μJ were 0.77% and 6.23%, respectively, and the RMSE and R^2^ for the predicted crater shape were 0.031 μm and 0.9, respectively. As depicted in Figure 12e,f, the experimental and predicted results for the diameter and depth of the ablation crater were 4.49 μm and 4.48 μm, and 0.36 μm and 0.41 μm, respectively, indicating that the errors of the ablation crater diameter and depth with a single-pulse energy of 2.2 μJ were 0.23% and 10.9%, respectively; the precision of the crater shape prediction was 0.032 μm in RMSE, and 0.91 in R^2^. Following the aforementioned verification, the model is capable of making predictions regarding the ablation morphology of GaN subjected to femtosecond laser ablation with a micron spot radius in a wide range of laser energies.

### 4.3. Ablation Threshold Prediction

**Figure 13:**
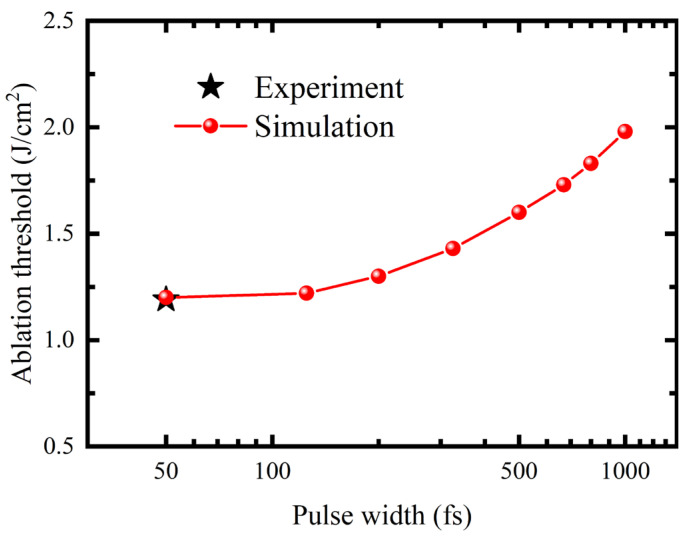
Ablation threshold at different pulse durations.

To provide a reference for the high-precision femtosecond processing of GaN, the threshold fluences for the single-pulse ablation of GaN with varying pulse widths in the range of 50–1000 fs were predicted on the basis of the isotherms, as illustrated in Figure 13. When the pulse width was 50 fs, the simulated threshold was quite consistent with the threshold measured in this experiment. A reduction in the ablation threshold was observed with a concomitant decrease in pulse duration within the range of 50–1000 fs. This phenomenon is attributable to the causal factor that the peak power exhibits an increase when the laser energy is maintained at a constant level and the pulse width is reduced. As a result, multiphoton ionisation becomes a more significant process, leading to a higher photo-ionisation rate and eventually a reduced threshold for ablation.

## 5. Conclusions

A multiphysics model of a single-pulse femtosecond laser with a micrometre-scale radius irradiating GaN was developed. In the simulation, the ablation morphology was proposed to be depicted by isothermal distributions. Experiments were performed to validate the model, in which GaN was ablated with various femtosecond laser single-pulse energies. It was found that the calculated isothermal distributions at 2660 K accurately predicted not only the ablation crater diameter and depth, but also the ablation crater profile across a broad range of laser fluences. The predicted errors of the ablation diameters and depths were within 4.71% and 10.9%, respectively. RMSE and R^2^ were employed to assess the prediction errors of the crater shapes, which fell within the range of 0.018–0.032 μm and 0.77–0.91, respectively. This study can provide an important reference to precisely conduct the femtosecond laser processing of GaN material with desired geometries.

It should be noted that the multiphysics model for the femtosecond laser single-pulse ablation of GaN established in this study is only capable of correlating single-pulse energies and ablation shape. The next step in our research will be to optimise the proposed model in order to accurately predict ablation results with different beam sizes, different incident laser fluence regime distributions, and different pulse numbers.

## Data Availability

The data presented in this study are available on request from the corresponding author. The data are not publicly available due to the need for further research.
